# Determinants to Continuation on Hiv Pre-exposure Propylaxis Among Female Sex Workers at a Referral Hospital in Uganda: a Mixed Methods Study Using Com-b Model

**DOI:** 10.21203/rs.3.rs-3914483/v1

**Published:** 2024-02-09

**Authors:** Samuel Kawuma, Rogers Katwesigye, Happy Walusaga, Praise Akatukunda, Joan Nangendo, Charles Kabugo, Moses R. Kamya, Fred C. Semitala

**Affiliations:** Makerere University Joint AIDS Program; Makerere University Joint AIDS Program; Makerere University Joint AIDS Program; Makerere University Joint AIDS Program; Infectious Diseases Research Collaboration; Kiruddu National Referral Hospital; Infectious Diseases Research Collaboration; Makerere University Joint AIDS Program

**Keywords:** PrEP continuation, Female sex workers, barriers, facilitators, determinants, Uganda, COM-B

## Abstract

**Background:**

Female sex workers (FSWs) have the highest HIV prevalence in Uganda. Pre exposure prophylaxis (PrEP) has been recommended as part of the HIV combination prevention strategy, with improved patient initiation, but continuation on the service is low. We evaluated PrEP continuation among FSWs and explored potential determinants of PrEP continuation within a public referral hospital in Urban Uganda.

**Methods:**

An explanatory sequential mixed method study was conducted at Kiruddu National referral hospital in Uganda. Secondary data on social demographic characteristics and follow up outcomes of at least one year was collected for all FSWs who were initiated PrEP between May 2020 and April 2021.We used Kaplan–Meier survival analysis to evaluate continuation on PrEP from time of initiation and follow-up period. The capability, opportunity, and motivation to change behaviour model was used to explore perspectives and practices of FSWs (n = 24) and health care providers (n = 8) on continuation on PrEP among FSWs, using semi structured interviews. The qualitative data was deductively coded and analyzed thematically, categorizing the themes related to PrEP continuation as facilitators and barriers.

**Results:**

Of the 292 FSWs initiated on PrEP during this period, 101 (34.6) % were active on PrEP, 137 (46.9%) were lost to follow-up, 45 (15.4%) were no longer eligible to continue PrEP, eight (2.7%) were transferred out and one (0.3%) had died. Median survival time on PrEP was 15 months (Interquartile range IQR, 3–21). The continuation rates on PrEP at six (6) and 12 months were, 61.1% and 53.1%, respectively. Facilitators of PrEP continuation included awareness of risk associated with sex work, integration of PrEP with other HIV prevention services, presence of PrEP Peer support and use of Drop-in centers. The barriers included low community awareness about PrEP, high mobility of sex workers, substance abuse, and the unfavorable daytime clinic schedules.

**Conclusion:**

Continuation on PrEP remains low among FSWs. Interventions for PrEP continuation should address barriers such as low community awareness on PrEP, substance abuse and restrictive health facility policies for scale of the PrEP program among FSWs in Uganda. Integration of PrEP with other services and scale up of community PrEP delivery structures may improve its continuation.

## Background

Data from Sub-Saharan Africa (SSA) show that 15% of new HIV infections occur among female sex workers (FSWs) [1], a patten similar to that seen in Uganda where HIV studies show a high HIV prevalence among sex workers at 37% compared to 5.5% in the general population [2, 3]. Innovative and effective HIV prevention interventions such as Oral pre-exposure prophylaxis (PrEP) with Tenofovir disoproxil fumarate coformulated with Emtricitabine or Lamivudine is recommended globally for preventing HIV acquisition in high risk groups [4–6]. Uganda adopted oral PrEP as part of the combination prevention biomedical intervention. However, the scale up of the PrEP program remains sub-optimal[7]. Whereas the acceptance of PrEP among FSWs has previously been established [8], there is a dearth of knowledge about the continuation on PrEP services including its determinants (facilitators and barriers) among FSWs. According to the Uganda annual joint AIDS review report, 90% of the FSWs found eligible were initiated on PrEP during 2021/2022 [9] but the trends on PrEP continuation are unknown. Previous studies conducted in Uganda cited PrEP stigma and high mobility as potential barriers to uptake and continuation on PrEP among high risk populations [10]. To inform the national PrEP program on the current continuation rates among eligible FSWs, we evaluated PrEP continuation rates among FSWs in a public national referral hospital. In addition, we explored the potential facilitators and barriers to continuation on PrEP services among the FSWs using the Capacity, Opportunity, Motivation to change behavior (COM-B) model of the behavior change wheel [11–13].

## Methodology

### Study design.

This was a mixed methods explanatory sequential design [14], where we retrospectively collected and analysed quantitative data on the social demographic characteristics and follow up outcomes of FSWs enrolled on PrEP first and later collected qualitative data to better understand and explain the findings of the quantitative data. We used quantitative data to determine the discontinuation rates on PrEP among eligible FSWs at various time intervals and the predictors of PrEP discontinuation. The qualitative data was then used to contextualise the quantitative findings, understanding the potential facilitators and barriers to continuation on PrEP among FSWs.

**Figure 1:**
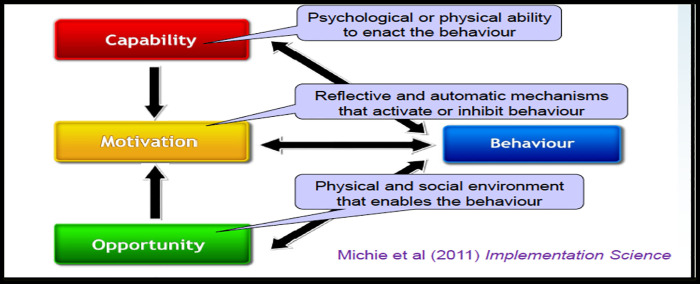
Illustration of the COM-B model

For the quantitative part, we collected data retrospectively on FSWs enrolled in PrEP at Kiruddu National referral hospital (KNRH) to map out the discontinuation rates and related factors. For the qualitative component, we purposively selected FSWs enrolled on PrEP and conducted semi structured interviews to explore their beliefs, understanding and perspectives on continuation on PrEP services. In addition, key informant interviews with health providers (HPs) at KNRH were conducted to share their experience and beliefs on continuation on PrEP services among eligible FSWs. We used the COM-B model, which postulates that for behavior change to occur, three conditions must be met: Capability, Opportunity, and Motivation (See Fig. 1) to explore the perspectives and practices of FSWs and health providers on continuation on PrEP among eligible FSWs.

### Study site.

The study was conducted at KNRH, a public tertiary level hospital located in the administrative division of Makindye of the Ugandan capital, Kampala. Makindye division is the largest of the five administrative divisions of Kampala with a high number of hotspots involved in sex work. KNRH provides HIV prevention care and treatment services at no cost to the patients including PrEP services to its catchment communities. At the time of this study a total number of 2,254 clients at substantial risk of HIV acquisition were active on PrEP, of whom 978 (43%) were FSWs. PrEP services at both health facility and community level are provided following the Uganda ministry of health guidelines. The PrEP services that include screening for eligibility, enrolment and follow up are offered by health providers including doctors, nurse counsellors, laboratory technicians, pharmacy technicians, data officers, and FSW PrEP peers with technical assistance by Makerere University Joint AIDS Program.

### Study participants and Sample size.

For the quantitative component of the study, we used a retrospective cohort study research design to collect quantitative data on the social demographic characteristics and follow up outcomes of FSWs who received PrEP services at KNRH between May 2020 and April 2021 and to allow for at least one year follow-up.

For the qualitative component we purposively selected a subset of 24 FSWs who were initiated on PrEP at KNRH, ≥ 18 years of age, about to return for PrEP refills and had been part of the quantitative study. To ensure diversity, both FSWs receiving PrEP refills at the facility and community based drop-in centres were included in the study. We recruited eight (8) health providers of different cadres (medical doctor, nurses, and Community-based FSW PrEP Peers) from the HIV outpatient clinic who participated in the provision of PrEP services at KNRH for at least one year.

Telephone calls were made to FSWs who were eligible to participate in the study and whose PrEP refill appointments were within two weeks. Health providers were approached physically at the facility and requested to participate in the study. The participants were recruited into the study until no new information was being obtained. In addition, the data provided was captured real time to know when data saturation was reached.

### Data collection.

For the quantitative component, secondary data was obtained from PrEP facility registers and the Health Management Information System (HMIS) AIDS Control Program (ACP) 027 that contain follow-up visit information. A data abstraction tool was developed to capture data of all FSWs who were initiated PrEP between May 2020 and April 2021 with their follow-up outcomes of at least one year as of April 2022. The baseline characteristics captured included age, date of PrEP initiation, education level, occupation, religion, PrEP continuation status, marital status, and place of residence and site of PrEP refills. The continuation on PrEP status was classified on whether the client was active on PrEP, lost to follow-up, transferred out, advised to stop PrEP by a health provider or died. Reasons for stopping PrEP by the health provider and transfer out were also captured. The extracted data was entered using Microsoft Access and later exported to Microsoft Excel for cleaning and coding. The data was analyzed with Stata version MP 14.0.

The qualitative study aimed to get a wider context of the possible enablers and barriers to continuation on PrEP services among FSWS. In-depth interviews with FSWs enrolled on PrEP and health providers involved in the provision of PrEP services were conducted. The pretested interview guides were developed based on the domains of the COM-B model and were administered by trained research assistants. 24 in-depth interviews were conducted with FSWs and eight Key informant interviews with health providers. Both sets of interviews lasted approximately 45 mins to one hour. We explored individual knowledge and beliefs about PrEP for HIV prevention, factors that influence decision to initiate on PrEP, how to receive the PrEP, the importance of PrEP and continuation on PrEP services. In addition, we generated individual lived experiences and perceptions of the proposed interventions to ensure continuation on PrEP services.

Written informed consent was obtained from all participants to join the study. For participants who could not read or write, informed consent was obtained in presence of an impartial witness. The interviews for health providers (HPs) were all conducted in English, audio recorded and later transcribed verbatim while those with FSWs were conducted in Luganda, the most spoken local language, audio recorded transcribed verbatim in Luganda and later translated in English.

### Data analysis.

Analysis of quantitative data involved the use of Kaplan–Meier survival analysis to evaluate discontinuation on PrEP from time of initiation, one month after initiation and every 3 months thereafter. The outcome variable was lost to follow-up, constructed as the time between initiation on PrEP and lost to follow-up (failure) as of April 2022. Lost to follow up was defined as no PrEP refill within one month after scheduled appointment.

Censoring was considered for individuals who continued PrEP, those who were no longer eligible for PrEP and stopped, transferred out and died within the study period. Our analysis considered the following as independent variables: age groups, education level, religion, marital status, residence, and site of PrEP refills. The Kaplan-Meier method was used to plot survival curves that served to test the proportional hazard assumption. We used the Cox’s Regression Hazards model to assess the association of the independent variables and PrEP discontinuation at 95% confidence interval. Stepwise analysis was used where covariates that had P-value ≤ 0.25 in bivariate cox regression analysis were selected for multivariable cox regression analysis. All the covariates with P-value ≤ 0.05 were considered to have statistically significant association with PrEP discontinuation during multivariable cox proportional regression analysis.

Analysis of the qualitative data involved de-identification of interview scripts which were later analyzed by the research team. The team comprised of a behavioral social scientist (HW) and public health specialist (SK) who analyzed data using a thematic content analysis [15, 16] guided by the six domains (physical/psychological capability, social/physical opportunity and reflective/automatic motivation) of the COM-B framework. The team developed a codebook by sampling some of the transcripts from the data. Each researcher independently identified preliminary codes and sub-themes using a deductive approach. Once a codebook was developed and the team agreed on recurring themes, HW reviewed all transcripts for completeness and later coded in Atlas ti qualitative analysis software version 22. The obtained themes were mapped to the COM-B domains and constructs and the data separated into facilitators and barriers that relate to continuation on PrEP services.

## Results

### Quantitative study.

#### Participant socio-demographic and baseline characteristics.

Between April 2020 and May 2021, a total of 292 FSWs were initiated on PrEP at KNRH with the baseline median age of 26 years, IQR, 22–30. (SD, ± 5.76). Majority (90.75%) received their PrEP refills from the community and were single (59.93%). (See Table 1).

#### PrEP discontinuation among Female Sex Workers.

Among the 292 FSWs who were initiated on PrEP during this period, 101 (34.6%) were still active on PrEP, 137 (46.9%) were lost to follow-up, 45 (15.4%) were stopped due to contraindications to PrEP, 8 (2.7%) were transferred out and one (0.3%) had died. Among those who stopped PrEP 42 (93.3%) were no longer at risk of HIV infection and three (6.7%) were due to other contra-indications to PrEP including low creatinine (< 60 mls/min), Hepatitis B positive, and unwillingness to continue PrEP by the client.

**Figure 2:**
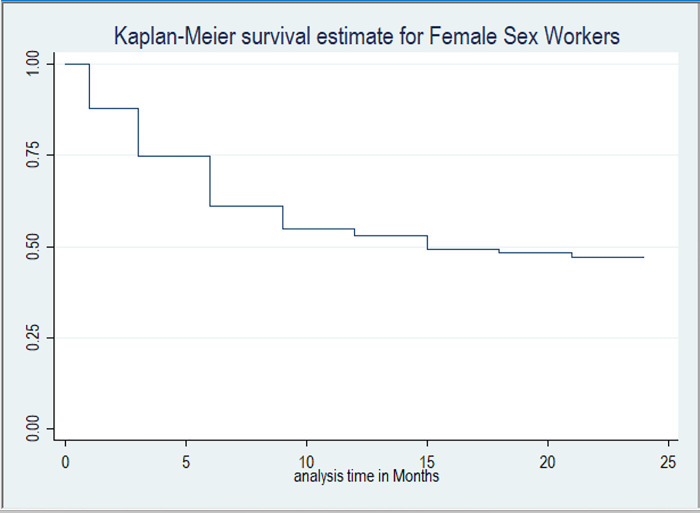
Kaplan-Meier survival curve for Female Sex Workers

Figure 2 shows the Kaplan Meier survival estimate for continuation on PrEP among FSWs at Kiruddu National referral hospital. The overall median survival time on PrEP was 15 months (IQR,3–21). The continuation rates on PrEP at one, three, six, nine, 12, 15, 18, 21 and 24 months were 88.0%, 74.7%, 61.1%, 54.9%, 53.1%, 49.3%, 48.4%, 47.1% and 47.0% respectively.

#### Factors associated with PrEP discontinuation among FSWs.

**Figure 3:**
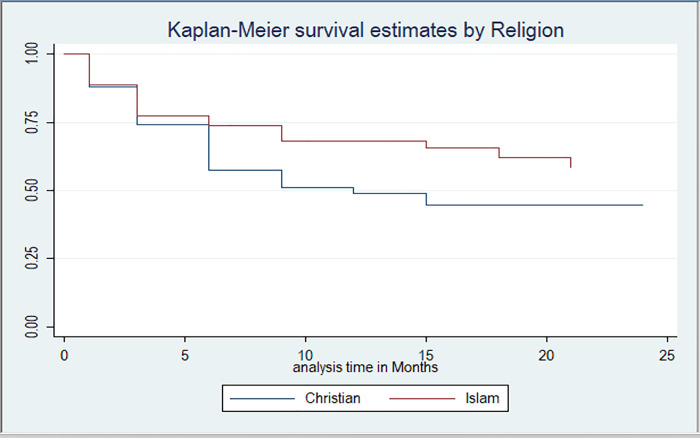
Kaplan-Meier survival curve for Sex Workers by Religion. (Log Rank Test: 0.039)

FSWs who were Christians had higher hazard rates of discontinuation at 56% compared to those that were Islam by religion (P-Value, 0.05, AHR = 1.56, 95% CI: 0.987, 2.467). (Table 2). There was no statistical relationship between PrEP discontinuation and age-group and education level. There was significant difference in discontinuation among FSWs by religion: Christians, Islam (Log Rank Test P-Value 0.039) (Fig. 3).

#### Integration of quantitative and qualitative findings.

The qualitative results below provide perspective to the perceived facilitators among health providers and FSWs on the continuation of PrEP among FSWs. The barriers and facilitators were presented along the domains of the COM-B model and explain why some FSWs stop or continue with PrEP services. Both barriers and facilitators emerged from the domains of the COM-B model including psychological capability, physical capability, physical opportunity, social opportunity, reflective motivation an automatic reflection. (Table 3).

### Qualitative Study

#### Participants.

A total of 24 FSWs aged ≥ 18 years receiving PrEP services at KNRH and eight (8) health providers offering PrEP services at KNRH participated in the study. The health providers included doctors (1), nurses (4), and two community health providers (PrEP Peers). Cumulatively, 24 in-depth interviews were conducted with FSWs and eight (8) Key informant interviews with health providers. Table 3 shows the perspectives of the health providers and FSWs as mapped on the COM-B model.

#### Perceived facilitators to continuation on PrEP services.

##### Psychological Capability.

FSWs expressed their understanding that sex work is associated with a high risk of HIV acquisition, which motivated them to continue PrEP services.

“What I understand by PrEP is, it is a medicine, that I use that can help prevent HIV/AIDs because as a sex worker, I have many sex partners.” FSW 013.

In addition, majority of the FSWs expressed the fear of death as an enabler to continue using PrEP.

“In this line of work (sex work), you have to encourage yourself to continue with PrEP, to avoid acquisition of HIV and death.” FSW 001.

Also, health providers had adequate knowledge on PrEP screening, eligibility criteria, and follow-up guidelines for PrEP which enabled the provision of appropriate information to PrEP recipients. In addition, they reported that most times, evaluation for PrEP services was also integrated with screening and management for other medical conditions such as sexually transmitted diseases which further enhanced continuation on PrEP services.

“PrEP is the use of ARVs to prevent the likely hood of HIV infection among high-risk HIV negative individuals. So, PrEP is taken as long as one continues to be at risk of contracting HIV.” HP 001-Counsellor

“When FSWs come here, we are able to offer PrEP, in addition, we also assess for other diseases, we offer general treatment, and this keeps them interested to come back” HP 002- Clinician.

#### Physical Opportunity.

Both health providers and FSWs acknowledged the role played by the supportive community structures in ensuring continuation on PrEP services. The presence of PrEP peer leaders at both the facility and established drop-in centers managed by the community-based organizations (CBOs) was a major facilitator. The peers supported in client mobilization, screening, and follow-up. In addition, the drop-in centers bring PrEP services closer to the target population including PrEP refills.

“I thank the management of drop-in centers (DICs) because, you may be stressed over a man who had sex with you, beaten you and not paid you, you come here, you are counselled, given something to eat, get your PrEP and bed to rest”. FSW 006.

“Like I said that we work with CBOs and peer leaders. All clients who are initiated at that facility are attached to peers who are key to their follow-up.” HP 006 -Counsellor.

#### Social Opportunity.

The health providers and FSWs believe that engagement of FSWs as peer supporters for continuation on PrEP services enables task shifting and relieves workload from the health providers. But also, the FSWs trust their peers to reach them in the community to provide PrEP refills.

“The peer model we use to mobilize FSWs in the hospital makes refilling PrEP easier. You find that you are alone, and you have over 300 clients to refill, it may be very hard to follow-up and refill these clients especially when some of them do not want to come back to hospital. So, the peer network and DICs have helped a lot “HP 005-Clinician.

“Sometimes the Peers bring us the medicine. when you fail to come back to the hospital. ” FSW 001.

#### Reflective Motivation.

Confidence and trust in the ability of the health providers to manage any side effects due to PrEP motivated FSWs to continue PrEP services.

“The doctors often call and check on us, we have that kind of relationship, they can even call you to remind you that your refill time is close and ask about any problems encountered with PrEP “. FSW 009.

Also, when FSWs are knowledgeable about the ability of PrEP to avert HIV acquisition, all of them are motivated to continue with PrEP services FSW 009.

“And most people say, “that girl is spoilt, she is a prostitute but in spite of that, she is HIV negative” but why is it so? This is because of PrEP” FSW 020.

#### Automatic reflection.

Most FSWs without children expressed their desire to establish a family as a motivator to continue PrEP services by being healthy while the ones with children wanted to stay negative to take care of their children.

“I must swallow PrEP because I am an orphan and still want to raise my children” FSW 024.

#### Female sex workers and health providers reported barriers to continuation on PrEP.

##### Psychological capacity.

Female sex workers lack adequate knowledge on PrEP medicine such as possible side effects were a barrier to continuation on PrEP services.

“Sometimes, I think the PrEP medicines stopped me from getting pregnant. I want to have a child, but it is not happening’ FSW 015.

Many FSWs felt there was inadequate community awareness about PrEP services. This requires them to give explanations to prospective customers, friends and family about PrEP and its benefits. The situation is worsened by the packaging that is like that of ARVs.

“Many people don’t know about PrEP, when they see the drug container, they may think they are antiretroviral therapy.” FSW 011.

“May be, the doctors need to further sensitize the people in the community about PrEP so that they can also be educated about it and begin to take it. They should go to different communities sensitizing people so that they can also benefit from the medication.” FSW 003.

#### Physical opportunities.

The majority of FSWs noted substance abuse as a key hindrance of retention on PrEP. The drugs such as alcohol interfere with their adherence to health worker instructions and subsequently impair continuation on PrEP services.

“I started drinking alcohol because of the kind of work I do. You may get a customer who first wants you to get drank. People are different so you may find one who wants to first have fun with you before sex.” FSW 010.

Both FSWs and health providers agreed that the increased workload at facility and long waiting time were structural barriers to continuation on PrEP services.

“The increasing number of clients on PrEP services with no increase in the healthcare work force impairs implementation of retention on PrEP strategies with fidelity” HP 004.

Moreover, majority of the FSWs expressed the lack of privacy and restrictive health facility policies such as dress code, centralized drug picking points (Pharmacy) as barriers found at the health facility.

“When you tell them to wait for the drugs at the pharmacy, some of them leave without the drugs. This affects retention efforts.” HP 001-consellor.

All health providers agreed that stock out of key commodities such as PrEP drugs and rapid HIV testing kits hinders their efforts to retain clients on PrEP services. This interferes with the conduct of standard follow-up activities such as repeat HIV testing and supply of multi month PrEP refills.

“We have challenges of commodity stock outs. Sometimes we have limited stocks and that affects our retention since we cannot dispense multi month refills which demoralizes the client.” HP 001.

“Stock-out of commodities such as rapid HIV testing kits deters health providers from reaching out to clients for their refills since you cannot perform PrEP refills before retesting the clients.” HP 008.

Female sex workers described long distances to facility or community refill points that increase transport expenses. They expressed the desire to have integrated services brought nearer to them through establishment of more community structures given their mobile nature and increase of multi-month refills to reduce hospital visits.

“Most times what hinders me from coming is transport. We move from one place to another; we therefore need more drop-in centers.” FSW 020

“I wish they (health providers) would put in place more community places within those far to reach areas and supply PREP, condoms and other prevention services. They should supply condoms, family planning injectable, and lubricants to the sex workers.” FSW 019

#### Reflective motivation.

Female sex workers felt that the attitudes of some health providers discouraged them from coming for PrEP refills. There was therefore a call for more safe spaces to offer services to the FSWs.

“Just the attention sex workers get when they enter the hospital drives them away. Everyone would be looking at them and they would feel out of place. We need places where we are more accepted”. FSW 004

## DISCUSSION

This explanatory sequential mixed methods study explored the trends on continuation on PrEP services among eligible FSWs as well as the barriers and facilitators of continuation on PrEP, using the COM-B model. There was high discontinuation of PrEP among FSWs with more than half-discontinuing PrEP within 15 months of initiation. However, in our cohort, early continuation on PrEP was high at 88.0% and 74.7% at one and three months respectively suggesting that the gaps are in the current strategies for long term continuation on PrEP among FSWs in Uganda. Similar high PrEP discontinuations among FSWs have been observed in other studies in sub-Saharan Africa[17, 18]. Therefore, our results suggest that there is need for more research on population specific interventions to sustain PrEP use overtime which is a critical issue in PrEP implementation[19].

Social demographic factors such as age and sex have been shown to affect continuation on PrEP services. In some studies, young users and being female were more likely to discontinue PrEP [20–22] compared to their adult and male counterparts respectively. Our study shades light on the possible role of religious beliefs and practices on persistence on PrEP services and hence the need for PrEP providers to explore PrEP recipient beliefs and address any concerns through comprehensive counselling. However, we did not find any significant association between age and continuation on PrEP services potentially because most of the study population was young.

Our qualitative study results emphasize the importance of improving PrEP knowledge among FSWs to ensure continuation on PrEP services. Understanding the use of PrEP, the side effects associated with PrEP would address stigma associated with PrEP use and mitigate PrEP discontinuations due to side effects as evidenced in South Africa [23]. In addition, PrEP programs should improve community awareness about PrEP services and address PrEP misconceptions such as PrEP recipients being mistaken for being on HIV treatment due to similarity in drug packaging [21]. Therefore, PrEP programs need to emphasize mass sensitization campaigns on PrEP for improved uptake, reduced stigmatization, and long-term continuation.

Consistence with evidence elsewhere [23, 24], our findings show that awareness of the risk associated with sex work was an enabler to continuation on PrEP services. Therefore, empowering people to accurately perceive their individual risk of HIV acquisition would lead to higher rates of PrEP continuation. Health providers should offer comprehensive education and counselling on sexual risk behavior to increase awareness and knowledge among female sex workers for long term continuation on PrEP services.

The presence of female sex worker Peers at both facility and community level was reported to enhance continuation on PrEP among FSWs and reduced workload on the health providers. The community health providers such as FSW Peers who are living through a similar experience provide psychosocial support, follow-up clients, deliver PrEP and improve client knowledge on PrEP [25].The Peers can in addition help to dispel myths, address misconceptions and provide accurate information about the benefits and importance of PrEP for female sex worker. This proactive Peer navigation has been shown to improve retention in HIV care and PrEP services and should be scaled up as we address the gap in persistence on PrEP among eligible populations [26–28].

In addition, both healthcare providers and FSWs emphasized the need for decentralized PrEP delivery through community structures such as the drop-in centers and community pharmacies to increase accessibility and continuation on PrEP services. Decentralization of PrEP delivery models enhances person centeredness and is key in demedicalisation of the services[29]. Through demedicalisation, facility level barriers to continuation on PrEP services shared by health providers and FSWs such as the increased workload, FSW adherence to a particular dress code, long waiting time during clinic visits and the frequent clinic visits also observed in other sub-Saharan countries such as Kenya [30] can be addressed.

Sex work is associated with high mobility [31] and our study results further emphasize its impact on FSWs adherence to PrEP refill schedules. Majority of the FSWs acknowledged that this occupational context of their work significantly influenced continuation on PrEP patterns. The findings underscore the quick adoption of six-month PrEP refills by the PrEP programs to reduce clinic visit frequency. Multi month drug refills as seen in HIV programs in SSA have improved retention in care among people living with HIV [32, 33] and the same approach could benefit PrEP programs. Also, the adoption of other PrEP options such as long acting cabotegravir in the PrEP program would address the pill burden barrier [34] and reduce clinic visits in this highly mobile population.

The study results showed how critical it is to integrate of PrEP services with other health care services such as family planning, substance use treatment, sexually transmitted treatment (STI) treatment, HIV testing and mental health support to enhance continuation on PrEP. The intersection of substance use and failure to return for PrEP refills reported by the FSWs emphasizes the importance of extra resources to enhance service integration in HIV clinics and primary care settings. PrEP programs should adopt the integrated approach to holistically address the needs of the clients, improves service access and utilization as evidenced elsewhere [35, 36].

Poor attitude of health providers towards FSWs and inconvenient clinic operation hours that impair access to prevention services were major barriers to continuation on PrEP services in this population. Most FSWs reported working during the night and rest during the day. However, health providers continued to schedule daytime appointments due to the inflexible clinic policies. Such discriminatory treatment of FSWs that may even involve denial of treatment after gender based violence and hostility from public sector health providers has also been found in studies conducted in Uganda, Kenya ,Zimbabwe and South Africa [37–39]. To address this problem, training and sensitization of public health care workers should constitute some of the multilevel interventions [38] aimed at ensuring the delivery of non-discriminatory, private and safe heath care services.

### Strengths and Limitations.

The use of multiple sources of data in the qualitative component (Health care providers, Female Sex Workers) and use of the COM-B model to guide data collection and analysis was a strength for the study [40].

The study was conducted in a single public health hospital offering PrEP services according to the Uganda national guidelines in an urban setting. Since the clinic is found in a national referral hospital, which receives clients from all over the country, we believe that the study findings are generalizable to other low-income countries.

### Conclusion

PrEP continuation rates remain low among FSWs. Integration of PrEP with other services, improved health provider knowledge on PrEP and scale up of community PrEP delivery structures will facilitate continuation on PrEP. In addition, interventions should address barriers to PrEP continuation such low community awareness on PrEP, substance abuse and health facility level barriers for the scale of the PrEP program among FSWs in Uganda.

## Figures and Tables

**Table 1:** Baseline characteristics of study Participants.

Variable	Total N = 292, %	Event n = 137 ,46.92	Censored n = 155, 53.08	P-Value
**Age**				
Median age (age group), SD	26 (22–30), ± 5.76	25(21–29), ± 5.72	27(23–30), ± 5.72	
**Age group**				
<25	113, 38.7	62, 45.3	51, 32.9	0.09
25–29	100, 34.3	41, 29.9	59, 38.1	
≥30	79, 27.1	34, 24.8	45, 29.0	
**Education level**				
None	83, 28.4	41, 29.9	42, 27.1	0.70
Primary	106, 36.3	49, 35.8	57, 36.8	
Secondary	97, 33.2	43, 31.4	54, 34.8	
Tertiary	6, 2.1	4, 2.9	2, 1.3	
**Religion**				
Christian	230, 78.8	115, 83.9	115, 74.2	0.04
Islam	62, 21.2	22, 16.1	40, 25.8	
**Marital status**				
Single	175, 59.9	89, 64.9	86, 55.5	0.55
Married	19, 6.5	8, 5.8	11, 7.1	
Separated	85, 29.1	35, 25.6	50, 32.3	
Divorced	9, 3.1	3, 2.2	6, 3.9	
Widowed	4, 1.4	2, 1.5	2, 1.3	
**Area of Residence**				
Central	35, 12.0	22, 16.06	13, 8.4	0.33
Kawempe	55, 18.8	26, 18.98	29, 18.7	
Kyadondo	15, 5.1	7, 5.11	8, 5.2	
Makindye	50, 17.1	24, 17.52	26, 16.8	
Nakawa	32, 11.0	11, 8.03	21, 13.6	
Rubaga	105, 36.0	47, 34.31	58, 37.4	
**Site of PrEP refills**				
Facility	27, 9.25	11, 8.0	16, 10.3	0.50
Community	265, 90.75	126, 92.0	139, 89.7	

PrEP - Pre-Exposure Prophylaxis; SD - Standard deviation

**Table 2:** Multivariable Cox Proportional Hazards Model.

Variable	UHR	P-Value	95% CI	AHR	P-Value	95% CI
**Age group**						
<25	Ref			Ref		
25–29	0.747	0.15	0.503–1.109	0.735	0.14	0.491–1.101
>30	0.823	0.36	0.541–1.251	0.787	0.27	0.516–1.206
**Religion**						
Islam	Ref			Ref		
Christian	1.561	0.06	0.988–2.466	1.560	0.05	0.987–2.467
**Education level**						
None	Ref			Ref		
Primary	1.037	0.87	0.684–1.571	1.052	0.81	0.693–1.596
Secondary	0.92	0.71	0.601–1.417	0.988	0.96	0.638–1.529
Tertiary	1.93	0.21	0.690–5.414	2.062	0.17	0.729–5.832

UHR - Unadjusted hazard ratio; CI - Confidence interval; Ref - Reference category.

**Table 3:** Facilitators and barriers to continuation on PrEP services in the Hospital as related to the COM-Bdomains.

C	Facilitators	Example quotes	Barriers	Example quotes
Psychological capability	**FSWs**:Awareness that sex work is associated with a high risk of HIV acquisition. Fear of death due HIV disease Experience of having friends who stopped PrEP and acquired HIV. **HP**: Healthcare providers have adequate knowledge of PrEP screening, initiation, and continuation.	“What I understand by PrEP is, it is a medicine, that I use that can help prevent HIV/AIDs because as a sex worker, I have many sex partners.”FSW 013. And most people say, “That girl is spoilt, she is a prostitute but in spite of that, she is HIV negative” but why is it so? Because of PrEP. And I tell them the same thing” FSW 020 PrEP is the use of ARVs to prevent the likely hood of HIV infection among high-risk HIV negative individuals. So, PrEP is taken as long as one continues to be at risk of contracting HIV. HP 001-Counsellor In this line of work (sex work) ,you have to encourage your self to continue with PrEP, to avoid acquisition of HIV and death.” FSW 001 *“When FSWs come here, we are able to offer PrEP, in addition, we also assess for other diseases, we offer general treatment, and this keeps them interested to come back” HP 002- Clinician.*	**FSWs** Misconceptions about PrEP medicine Inadequate knowledge on how to manage side effects associated with PrEP. Inadequate community awareness about PrEP services. Poor health worker attitudes towards FSWs,	“Sometimes the medicine I think stopped me from giving birth. I want to have a child, but it is not happening’ FSW 015. “May be, the doctors need to further sensitize the people in the community about PrEP so that they can also be educated about it and begin to take it. They should go to different communities sensitizing people so that they can also start their medication.” FSW 003.
Physical capability	None.		None.	
Physical opportunity	**FSWs** Presence of health education ,support and monitoring by health providers Supportive infrastructure at both facility and community including the PrEP Peer leaders. Integration of other services during the PrEP refill visits. Availability of PrEP drugs. Easy accessibility of PrEP services. **HPs**: Flexibility of work ensures clients retention	“I will thank the management of drop-in centers (DICs) because, you may be stressed over a man who had sex with you, beaten you and not paid you, you come here, you are counselled, given something to eat and bed to rest”.FSW 006. “The medicines are available and sometimes they bring us the medicine when we fail to come. “ FSW 001 **HPs** “Then we have flexi hours clinics. So, those who are unable to come for refills during the weekdays can be refilled on Saturday or the evening” HP 001- Counsellor “Like I said that we work with CBOs and Peer leaders. All clients who are initiated at that facility are attached to Peers who are key to their follow-up”HP 006- Counsellor	**FSWs** Substance abuse/Alcohol consumption The daytime clinic schedules are not in sync with the daily routines of sex work. Insufficient commodities at the facility such as PrEP drugs and HIV testing kits Increased workload by health providers. Limited Privacy at the hospital. Restrictive health facility policies such as dress code. High mobility from one place to another The busy work schedules . The burden of swallowing the pill every day. Long distance to facility or community refill points, long waiting times at the clinic due to routine assessments **HPs**: Time consuming data collection tools Stock out of supplies such as rapid testing kits, drugs for sexually transmitted infection (STI) treatment and PrEP, discourages FSWs from returning. This also limits MMDs in PrEP programming.	“The other thing that’s so common among the sex workers, they want to sleep during the day. We want everything (clinical) to be done in the night, yet the doctors are readily available during the day.”FSW 009 “Most times what hinders me from coming is transport, we need more drop in centers.” FSW 020 “I started drinking alcohol because the kind of work I do. You may get a customer who first wants you to get drank. People are different so you may find one who wants to first have fun with you before going to bed”. FSW 010. “When you tell them to wait for the drugs at the pharmacy, some of them leave without the drugs. So that one is also affecting us.”HW 001- Consellor. The increasing number of clients on PrEP services with no increase in the healthcare work force impairs implementation of retention on PrEP strategies with fidelity” HP 004. “We have challenges of commodity stock outs. Sometimes we have limited stocks and that affects our retention since we cannot dispense multi month refills which demoralizes the client” HP 001. “Stock-out of commodities such as testing kits (Determine) deters health providers from reaching out to clients for their refills since you cannot perform PrEP refills before retesting the clients” HP 008.
Social opportunity	**FSWs**: Disclosure to a trusted relative/friend. Peer attachment at facility or community **HPs**: Task shift to peers based at the facility who carry out some tasks when the health providers are overwhelmed with workload.	“…..But some friends encourage me and tell me to be strong because life is mine, take the medicine, after all you are not taking it for HIV. When God gives you marriage, you will stop this job. But just take your medicine.” FSW 006. The Peer model we use to mobilize them in the hospitals makes at least refilling PrEP easier. You find that you are alone, and you have over 300 clients to refill, it may be very hard follow and refill these clients especially when some of them don’t want to come back to hospital. So, the peer network and DICs have helped a lot ’.’ HP 005-Clinician	Nil	Nil
Reflective motivation	**FSWs** Confidence and trust in the ability of health providers to manage any PrEP side effects. Understanding that indeed PrEP prevents HIV acquisition if continued with by FSW. **HPs**: Regular reports on retention keep health providers updated.	**HPs** “We have systems like weekly, monthly and quarterly reports. All those reports I think are the systems that enable retention”.HP 006. “The doctors often call and check on us, we have that kind of relationship, they can even call you to remind you that your refill time is close and ask about any problems encountered with PrEP. This has helped us to always pick the medicine in time and since I am in this business (Sex work), the message that HIV/AIDS has no cure often rings a bell in my mind and it is me that has the power to prevent it”. FSW 009.	**FSWs** Attitudes of some health providers may discourage FSWs from coming for PrEP refills	“just the attention sex workers get when they enter the hospital drives them away instead. Everyone would be looking at them and they would feel out of place ” FSW 004.
Automatic motivation	**FSWs** Desire to establish a family.	**FSWs** ’ I must swallow PrEP because I am an Orphan and I still want to raise my children” FSW 024.	Nil	Nil

FSWs - Female sex workers; PrEP - Pre-exposure prophylaxis; HIV - Human immunodeficiency virus; ARVs - Antiretroviral Drugs; HPs - Health Providers; MMD - Multi-Month Dispensing; HCW - Health Care Worker, DICs - Drop in centers; CBOs - Community-based organizations,STI- Sexually transmitted infections.

## Abbreviations

ART: Antiretroviral therapy
FSW: Female sex worker
HIV: Human Immune virus
KNRH: Kiruddu national referral hospital
MJAP: Makerere university Joint AIDS Program
MOH: Ministry of health
UAC: Uganda AIDS commission
PrEP: Pre exposure prophylaxis
PEP: Post exposure prophylaxis
PLHIV: People living with HIV
CDC: Center for disease control and prevention
WHO: World health Organization
HP: Health provider
